# Australian key stakeholder views regarding implementation of atrial fibrillation screening: a qualitative evaluation

**DOI:** 10.1136/bmjopen-2025-109404

**Published:** 2026-05-20

**Authors:** Kirsty Mckenzie, Ben Freedman, Anushka Jacob, Rakesh Narendra Modi, Nicole Lowres

**Affiliations:** 1 Heart Research Institute Ltd, Newtown, New South Wales, Australia; 2 Charles Sturt University, Albury, New South Wales, Australia; 3 The University of Sydney Faculty of Medicine and Health, Sydney, New South Wales, Australia; 4 University of Cambridge Primary Care Unit, Cambridge, UK

**Keywords:** Mass Screening, Pacing & electrophysiology, QUALITATIVE RESEARCH, Implementation Science, Primary Care

## Abstract

**Abstract:**

**Objective:**

To understand the issues impacting atrial fibrillation (AF) screening and what needs to be considered for a successful national screening programme in Australia.

**Design:**

Qualitative design using semistructured interviews and thematic analysis.

**Setting:**

Australian Health.

**Participants:**

Six broad stakeholder groups were identified: charities/patient support, healthcare providers, professional bodies, government, research (including Indigenous health) and industry.

**Methods:**

Semistructured interviews were conducted with 25 representative participants. Iterative thematic analysis was used. Coding was driven by the research questions (the current context; is a national screening programme warranted and approaches to a national screening programme) and an inductive approach where novel groupings of information were identified.

**Findings:**

The key findings are grouped into four areas. (1) Current opportunistic general practitioner-led screening is ad hoc and fragmented. Issues: poor remuneration; lack of health sector collaboration and prioritisation; consumers lack awareness. (2) Systematic screening of all in-scope patients not considered feasible and concerns over lack of evidence. (3) Alternative approaches to increase screening include innovative approaches inside and outside general practice and use of smart technology. (4) Recommendations: (a) Support general practices and address remuneration and workflows; (b) Ensure a clear pathway to treatment; (c) Address data security, management and integration and sensitivity issues with wearable devices; (d) Promote collaboration between key organisations; (e) Address research gaps and (f) Generate culturally appropriate consumer education to promote consumer demand.

**Conclusions:**

Most stakeholders were broadly supportive of AF screening but agreed that current approaches were fragmented and not sufficient. If the forthcoming research evidence supports screening effectiveness on major outcomes, stakeholders envisaged a semi-systematic approach tailored to specific health settings, rather than a formalised systematic national screening programme.

**STRENGTHS AND LIMITATIONS OF THIS STUDY**

- A key strength of this research is that it involves a wide range of stakeholders from policy-makers to clinicians and charity groups.
- Relevant stakeholders were identified through extensive stakeholder mapping.
- The exploratory design used iterative thematic analysis, and consideration was given to the importance of all deviant cases, so that themes were not simplified to the commonly held views.
- Views may not fully represent all of Australia, which is diverse in culture and rurality and has a complex multitiered system of healthcare governance, as our findings are limited to the views of invited stakeholders who agreed to participate, and we did not have representation from every region and level of government.

## Introduction

 In Australia in 2023, there were 28 252 first-ever ischaemic strokes, costing ~US$38 000 for the first 3 months after each stroke.^1^ Approximately 30% of these strokes are associated with atrial fibrillation (AF).^2^ Early identification of AF and appropriate treatment can reduce the risk of a stroke by 64%.^3^

Australian and European AF management guidelines both recommend opportunistic screening of adults aged 65 and over, using either pulse palpation or single-lead ECG.^4 5^ The Asia Pacific Heart Rhythm Society also published AF screening practice guidance outlining multiple methods for screening.^6^ Globally, multiple strategies for implementation of screening have been researched,^7^ with many demonstrating cost effectiveness.^8^,^10^ However, despite evidence and guideline recommendations, only 10%–15% of general practitioners (GPs) in Australia routinely screen opportunistically.^11^

Qualitative research on AF screening has mainly focused on acceptability of specific screening interventions, rather than screening as a whole. Therefore, little is known about Australian key stakeholders’ perspectives regarding the implementation of AF screening in Australia. Understanding these perspectives would identify issues that need to be addressed before a national screening programme can be considered. Therefore, this study sought to answer the following research questions:

1. What issues operate in the current context that are impacting effective screening for AF?
2. Is a national screening programme warranted? What needs to be addressed before considering a programme?
3. How could a national screening programme operate in the Australian context? What issues need to be addressed for a national screening programme to operate successfully?

## Methods

### Study design

This study used an exploratory research approach using qualitative analysis of semistructured interviews.

### Participants

**Figure 1:**
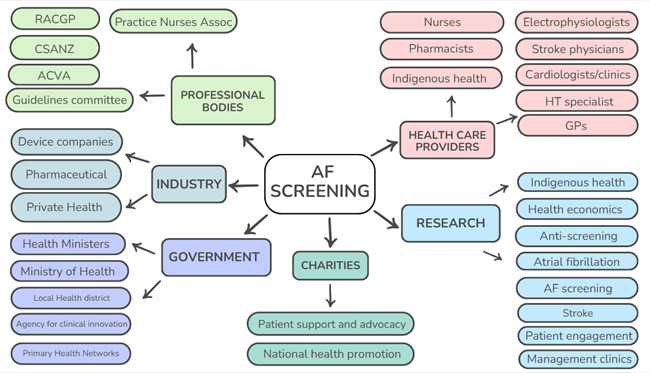
Stakeholder mapping. ACVA, Australian Cardiovascular Alliance; AF, atrial fibrillation; CSANZ, Cardiac Society of Australia and New Zealand; GP, general practitioner; HT, hypertension; RACGP, Royal Australian College of General Practitioners.

Relevant stakeholder groups pertinent to AF screening were identified through stakeholder mapping, based on preliminary work performed in the UK for the SAFER (Screening for Atrial Fibrillation with ECG to Reduce stroke) study, and adapted to the Australian health setting (figure 1). Six broad groups were identified: charities/support groups, healthcare providers, professional bodies, government, research and industry with 31 subgroups in total (figure 1). A purposive sampling approach was used. Organisations from the identified stakeholder subgroups were approached and asked to suggest relevant personnel to be interviewed. Invitations were emailed to stakeholders from each of the 31 subgroups. We continued to invite stakeholders until we had adequate representation across the six broad stakeholder groups. In total, 45 stakeholders were invited and 25 agreed to participate. All participants provided written informed consent.

### Data collection

Semistructured interviews were conducted with 25 participants. Interviews were conducted over Microsoft Teams, between August 2023 and March 2024. Interviews ran for approximately 50 min and were recorded with participant consent. A semistructured interview guide was used (online supplemental file 1) based on a guide originally developed by the qualitative team for the SAFER trial in the UK. Participants were asked about their views on what is currently working and not working in the detection of AF in Australia, and aspects of a national screening programme including barriers, facilitators, benefits and harms.

### Analysis

The interviews were transcribed verbatim and analysed using an iterative thematic analysis approach.^12^ Coding was undertaken by KM. Coding was driven by a pre-existing framework derived from the research questions and an inductive approach in which novel groupings of information were identified from the data.^13^ Each transcript was coded line-by-line based on identifying similar concepts, ideas and patterns in the data, and concerning the research questions. Attention was given to whether deviant cases (uncommonly expressed ideas) might be important. Once all transcripts were coded, the research team met to discuss the working coding structure and group these into themes. To ensure that results reflected participants’ views and were not overly simplified, emerging themes were checked against the transcripts. Various measures were taken to reduce bias. During the initial coding phase, a subset of transcripts was also read by NL, and KM and NL met to compare their response to the data and refine the coding structure. KM also kept notes on her responses to participants' perspectives, which in cases differed from her own so these could be taken into account during coding (see online supplemental file 2: Reflexivity statement). After themes were finalised, they were developed into a narrative, which both explained the themes and provided evidence from the data in the form of quotations.

### Patient and public involvement

A patient representative was involved in reviewing the content of the interview guide. This study was about non-patient stakeholders, so deeper involvement of patients or the public was not highly pertinent.

## Results

We recruited 25 participants representative of six broad stakeholder groups (figure 1). All participants were considered experts, with at least 10 years’ experience in their current position, and many had experience in several professional roles and could consider AF from different perspectives. Participants were given a participant number identifying their stakeholder group (table 1). Seven participants were in more than one group.

**Table 1:** Participants by stakeholder group

Stakeholder group	Identifier	Participant number
Healthcare providers (GP, cardiologist, nurse; electrophysiologist, pharmacist)	**H**	H/R-04; H-05; H/P-09; H/R-11; H-12; H/G-17; H/R-18; H/P-19; H-21
Professional bodies	**P**	P-08; H/P-09; P-14; H/P-19
Government	**G**	G/R-13; G-16; H/G-17; G-22
Charities/support groups	**C**	C-02; C-03; C-06; C-07; C-10
Research (including Indigenous health)	**R**	H/R-04; H/R-11; G/R-13; H/R-18; R-20; R-23; R-24; R-25
Industry	**I**	I-01; I-15

GP, general practitioner.

**Figure 2:**
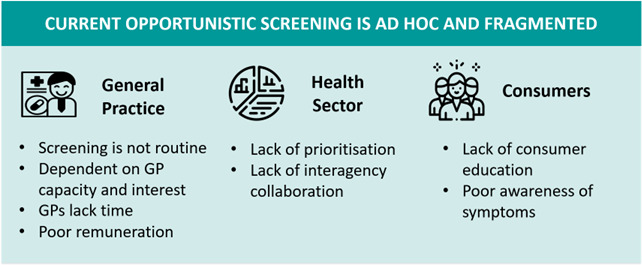
Issues related to current screening approaches. GP, general practitioner.

**Figure 3:**
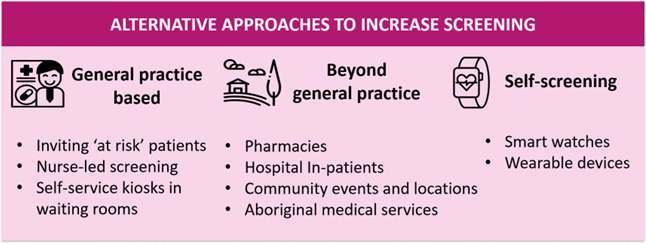
Screening approaches beyond the general practitioner.

**Figure 4:**
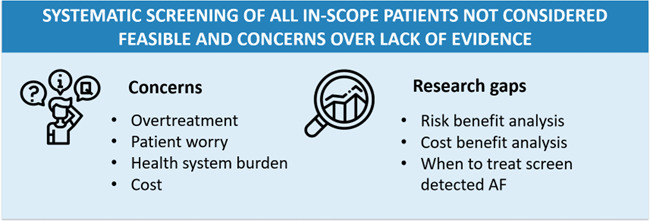
Concerns related to systematic screening. AF, atrial fibrillation.

Analysis identified key themes organised around the three research questions: the current context; is a national screening programme warranted; and approaches to a national screening programme. Themes for each question are discussed below and represented in figures 2–4. In analysing the data, we were attentive to differences in perspective between stakeholder groups. However, for most findings, there was broad agreement between groups on the existing challenges, needs and potential solutions. A decision was therefore made to not differentiate findings systematically by stakeholder group but to make reference where appropriate to views that were expressed more strongly by specific stakeholder groups.

### The current context

#### Opportunistic screening is ad hoc and fragmented and dependent on GP capacity

Participants described the current detection of AF in Australia as ‘ad hoc’ and ‘fragmented’ (I-01; C-02; C-03; C-06; G-16). Current detection of AF was seen as dependent on the patient recognising symptoms and going to the GP, or on the capacity of individual GPs to screen opportunistically as per the guidelines (I-01; C-02; C-03; C-06; C-07; P-08; H/P-09; H/R-11; G/R-13; P-14; I-15; G-16; H/G-17; G-22; R-24; R-25). However, participants from all stakeholder groups expressed concerns about undetected AF in the general population, and there was agreement across all stakeholder groups that many GPs were not opportunistically screening (I-01; C-02; H-05; C-06; C-07; P-08; G/R-13; P-14; H/P-19; H-21; G-22; R-25). This was also an issue in Aboriginal Medical Centres [R-25]. Lack of patient understanding and awareness of AF and its symptoms compounded this problem [C-06; C-07; H/P-09].

C-02 commented that the success of opportunistic screening was *“…kind of almost up to the GP and how their practice works and whether or not they have a personal interest” [C-02],* and several GPs described prioritising AF screening because of involvement in a research project or personal experience [H/P-09; H/G-17]. However, many GPs might not prioritise guideline-recommended screening of asymptomatic patients because they lacked awareness, time within the consult or had other priorities. This was recognised not only by GPs and other healthcare professionals, but was also by participants in other areas (I-01; C-07; P-08; H/P-09; H/R-11; G/R-13; P-14; I-15; G-16; H/G-17; R-24; R-25). *“They will forget, they will get busy, they will not do it… I 100% do not believe it’s a motivational issue or a lack of caring issue. It’s you know they had 12 complaints when they came in and the 15 minutes went really fast” [R-24]*. The business model of healthcare (general practice and pharmacy) impacted on capacity to screen for AF (C-02; H/R-04; H-05; C-07; H-12), and AF screening was seen as being inadequately remunerated (C-02; C-02; H-05; C-06; C-07; P-08; H/P-09; P-14; G-16; R-23).

#### Access to medical services

Compounding these problems was the issue of uneven access to medical services including GPs and cardiologists, based on socioeconomic factors and/or lack of services (especially in regional and remote areas), and participants from all stakeholder groups had concerns that patients who might benefit from being screened might not have access to a GP, and/or a cardiologist if required (I-01; C-02; C-03; H/R-04; C-06; P-08; H/P-09; C-10; H-12; G/R-13; H/G-17; R-23). Even in urban areas, access to medical services was perceived as not as good in disadvantaged areas, and people with lower incomes may not be able to access services (R-23).

#### Collaboration and prioritisation

The wider healthcare field was seen as fragmented and lacking effective inter-agency collaboration and resource sharing (figure 2) (C-03; C-07; P-14; I-15; H/R-18). Many organisations have ‘their own agenda because they are all in competition with each other’ [C-07], and unfortunately AF sat between key organisations and was ‘falling between the gaps’ [C-03]. In addition, there is a lack of prioritisation of AF by many organisations (C-02; C-06; C-07; H-12; P-14; I-15; H/R-18; H-21). While these organisations might ‘want to do more around the AF space’, there were insufficient resources to do ‘everything’ (C-03; H-21). Promoting collaboration (eg, through the Australian Cardiovascular Alliance) was seen by some as essential to create change (C-06; P-14; H/R-18; R-20), and the sector is ‘getting better’ in this regard (R-20).

### Is a national screening programme warranted?

#### Benefits and harms

Many participants believed there was value in screening for AF, and mentioned various benefits of a national screening programme (I-01; C-02; C-03; H-05; C-06; P-08; H/P-09; G/R-13; I-15; H/G-17; H/P-19; R-20; H-21; G-22; R-23; R-24). These included: potential to reduce stroke; detection of ‘hidden’ AF; incidental detection of other health issues; opportunities for lifestyle modification which have benefits beyond reducing burden of AF; and increased awareness in the community. However, almost all participants mentioned at least one potential harm, although the relative importance of this varied. Harms included increasing burden in the healthcare system, patient worry and harms associated with anti-coagulants. Some described the potential for false positives (especially from smartwatches) to ‘overwhelm the health system’ (I-01) and create unnecessary patient worry (G/R-13; I-15; H/P-19; R-20). However, for some, a false positive was better than ‘an unknown positive’ (H-05), and that potential for worry was outweighed by the benefits of stroke prevention (I-01; C-02; C-03; H-05; R-24).

As screening can detect large numbers of low-risk individuals, there was considerable concern surrounding ‘what counts as clinically significant AF?’ and when to treat with anticoagulants? (I-01; H/R-04; P-08; H/P-09; H-12; G/R-13; I-15; H/G-17; H/R-18; R-20; H-21; G-22). This was a key concern for healthcare providers and those working in research, one of whom commented that “the jury is out [regarding who should be given anticoagulants]” [H/R-18]. Consequently, screening could lead to overtreatment with anticoagulants, as well as burden the healthcare system and create worry for individuals (H-12; G/R-13; I-15; H/G-17; H-21; G-22). However, these concerns might not apply to all patient groups. As Indigenous Australians have a higher burden of cardiovascular disease, even ‘a little bit of AF is a big problem’ in this population and should be treated [R-25].

#### The need for evidence to justify a national systematic screening programme

There was very little support for a systematic screening programme (eg, through a national mailout to all citizens over the age of 65). Participants from all stakeholder groups saw this approach as not cost effective or as unjustified without a cost–benefit analysis and as creating burden for GPs, with money better allocated to encouraging GPs to engage more with opportunistically screening patients (H-05; H/P-09; C-10; H/R-11; G/R-13; P-14; I-15; G-16; H/G-17; R-20; H/P-21). There was also a need for evidence on reduction in stroke (systematic screening vs opportunistic screening/clinical detection) (H/P-09; H/P-19). One participant mentioned a randomised controlled trial that was in the early roll-out phase, commenting that “it will be interesting to see how many get picked up and how much of a difference it makes”, given that the methodology employed was “far more expensive and intrusive” for patients compared with opportunistic screening by a GP [P-09]. Another concern that was strongly expressed by researchers and healthcare professionals was the need for a risk–benefit analysis (including potential harms associated with anticoagulation) (G/R-13; H/R-18; H/P-19; H-21).

[Research needs to] evaluate the potential benefits and harms. I think that is really important. Particularly as you move more to the screening people that [have a] milder spectrum of disease or lower risk [of outcomes from untreated AF]. The potential for harm is still there. And the potential for benefit becomes smaller and more uncertain. [You] really do need to have good, robust evidence, ideally from randomised controlled trials, if you are wanting to roll out a screening test across a large number of people in the population [G/R-13].

Risk–benefit analyses also need to consider how AF is managed once detected, including a clear accessible evidence-based treatment pathway. Several healthcare professionals and researchers expressed concerns about anticoagulation treatment, given the potential risks (H/R-18; H/P-19; H-21) and medication compliance issues (R-23), and argued that management strategies should also consider ablation and management of risk factors including obesity (H/R-18; H/P-19; H-21; R-25). One participant argued that current data did not support anticoagulation for screen-detected AF, but that “in terms of slowing disease process and potentially reversing disease process, there’s good evidence for risk factor management… and I think that’s why it’s going to be important for us to keep screening” [H/R-18]

### Approaches and issues for a national screening programme: who, where, how?

Participants from all stakeholder groups made suggestions for ways to improve screening of AF in Australia. There were differing ideas about the practicalities of how a National Screening Programme should operate, including (1) who should perform screening; (2) remuneration solutions; (3) where screening should occur and (4) what devices to use. When asked what a more systematic, national approach could look like, participants made a wide range of suggestions. It is worth noting that these tended to be approaches that would increase opportunistic screening, either in general practice or in the community, rather than approaches that were truly systematic.

### General practice-based screening

The only genuinely systematic approach identified was inviting all people≥65 years to attend their GP for screening, via a national mailout. However, as noted above, there was limited support for this approach without justificatory evidence, and recognition that GPs did not have capacity.

Alternate solutions within general practice which reduced the need for GPs to screen were preferred, especially by healthcare practitioners, who favoured screening by practice nurses during care plan consultations, influenza vaccinations or 75+Health Assessments (H/R-04; H/P-09; H/R-11). AF screening could be incorporated in the Heart Health Check (P-14); however, commentary highlighted poor uptake of the Heart Health Check, with suggestions its remuneration structure disincentivised GPs. Furthermore, AF and the Heart Health Check were thought to be ‘mutually exclusive in terms of age groups’ (I-15). Other solutions included patient-led screening in waiting rooms using self-service kiosks.

Another suggested alternative to screening all adults ≥65 years was screening ‘high risk’ patients (eg, high AF risk or additional stroke risk factors) (H/R-11; I-15; G-16; H/G-17; H/R-18; H/P-19). Targeted screening was seen as conserving resources and getting ‘the best bang for your buck’ (H/G-17). H/G-17 commented that everyone on a care plan should be given an ECG because “they’re all your higher risk patients anyway”.

Remuneration was mentioned by most participants. There is no current remuneration for AF screening under the Medicare Benefits Schedule. Medicare item numbers identify specific medical services or diagnostic procedures that are eligible for a government subsidy. This would mean the cost of screening would be covered by the government rather than the patient. Some participants, including GPs, discussed problems with Medicare in the context of screening, and/or mentioned the need for a new Medicare item number (H-05; C-07; P-08; C-10; G-16; H/G-17; H-21). However, one GP commented this was not in reality a barrier for AF screening, and Medicare item numbers are complicated enough without adding another one (H/P-09). Practice Incentive Payments (PIPs) for increased screening at a practice level were suggested; although H/G-17 mentioned PIPs did not work very well, and a Medicare item number would be more appropriate. PIPs are paid to the practice and are time-limited. Often, the practice will have systematised the intervention by the time payments are received, although this is not always the case (H/P-09). An effective GP model could include remuneration via Primary Health Network (PHN) commissioning to individual practices to screen for AF (G-16). Using this model, PHNs could assist via a local support team.

While some suggestions could help reduce GP burden, they rely on capacity at a practice level. Several participants (H/P-09; G-16, H/G-17, R-24) noted some practices might not have capacity for such programmes, even with additional funding and assistance. These included solo GPs, non-accredited practices and non-digitised practices.

#### Beyond general practice

Given the potential burden on GPs of a screening programme, there was a widespread agreement that avenues other than primary care could be incorporated (C-02; C-03; H/R-04; C-07; C-10; H/R-11; G-16; H/P-19; H-21; R-24). These included screening in hospital; a greater role for pharmacy; community locations (eg, hairdressers or barbers); and public events where trained professionals take a single-lead ECG (figure 3). However, in these scenarios, a clear pathway to treatment was highlighted for suspected AF cases to receive appropriate follow-up with a medical professional for diagnosis and treatment.

#### Aboriginal medical services

It was argued that Aboriginal communities should be screened for AF at a younger age, due to a higher burden of AF and ill health at an earlier age (R-23; R-24; R-25). Aboriginal medical services (AMS) were seen as a logical setting for screening (R-23; R-24; R-25). AMS provides health services to local indigenous communities, using indigenous and non-indigenous doctors and nurses, and aboriginal health workers who could screen. However, not all people attend AMS, so any solution in these communities would also need to include a screening programme in non-AMS general practices (R-23; R-24).

Some AMS run unique clinics which could facilitate AF screening. Community members are invited to come and be screened for various issues (eg, diabetes). These are seen as ‘a day out’ where people can bring their family and get a meal (R-23).

AMS faces similar issues to other medical settings, as staff are busy and adding extra tasks may not always be feasible (R-24; R-25). Implementation would require strategies to overcome complex barriers related to AF-specific training for all AMS staff: especially short-term locum doctors who may not understand AF burden in aboriginal communities (R-24); and training sufficient Aboriginal health workers (R-25). In addition, at a practice level, AMS vary in their capacity to engage with funding programmes to increase screening rates and thus may require help with reporting requirements to apply for and obtain funding (R-23).

#### Self-screening

Wearables and smart watches were seen as a great opportunity to increase screening (figure 4) (C-02; C-03; H/R-04; C-07; H/R-18). “*What kind of frustrates me is the GP is the panacea to everything, when companies like Google and Apple and other industry partners are quite advanced in the technological and business model of screening and integration*” [H/R-04]. It could be possible to ‘just give everyone an Apple Watch’ [C-07]. However, participants identified problems with smart technology, including data management and security; linking patients with care providers; varied sensitivity and potential for false positives; the clinical relevance of device-detected AF; and relevance of the technology to the cohort (I-01; C-02; H/R-04; P-08; H-12).

### Software, data management and interoperability of systems

Software solutions and data management were seen as integral in AF screening solutions. Screening prompts via a pop-up tab or dashboard were described as valuable (H/P-09; R-23). However, there are *“complexities in electronic decisions, software and fatigue when it comes to alerts and triggers.”* [H/R-04]

Many screening solutions require transfer of data, raising issues of data security, especially for approaches utilising commercially available wearable devices such as smartwatches (H/R-04). Therefore, we need to consider how to *“best integrate them into the health system? It’s fraught with issues around data security and how you can integrate those things into clinical practice as well”* [H/R-04]. Currently, data are stored on the watch/device, and it is the patient’s responsibility to act on this data [I-01].

Effective interoperability between systems is crucial. It is essential to understand how data could be *“transferred into the patient file in an effective way…without crashing the server”* [R-25]. Currently, transferring relevant data into patient records can be *“cumbersome”* [R-24], and *“there is a chunk of IT software data transition issues”* [R-25]. The digital environment in which screening takes place is important to consider at a practice level, including lead times for organisations to integrate programmes into their software; and how these fit within practice workflow [C-10].

### Consumer awareness and education

There was broad consensus that increasing consumer awareness would benefit any screening programme. Some participants perceived that consumer awareness of AF is generally low, with some suggesting that health literacy is limited in certain communities (C-10; H/P-19; R-23; R-24). Increased consumer knowledge was seen as beneficial and would reduce the time spent by GPs in a consult. Participants from all stakeholder groups argued that patients needed to take responsibility for their own health, and consumer education could promote this (I-01; C-02; C-03; H/R-04; H-05; C-06; C-07; H/P-09; H/R-11; R-23; R-24). For example, in regional/remote Aboriginal communities, women who were educated about AF could facilitate screening by encouraging community members to visit their GP to be screened, or ‘harangue’ their doctor to screen (R-23; R-24).

Understanding diversity of consumers is important when providing education. Culturally appropriate communication materials are needed for messaging to reach all audiences. In Aboriginal communities, messaging regarding lifestyle changes, such as giving up smoking, emphasises it is “not just about you, it’s about us [the wider aboriginal community]” [R-23]. There was also a need to explore how best to communicate with Culturally and Linguistically Diverse communities (C-10; R-20). There were benefits to showing ‘the faces of AF’ [P-14] or using personal stories, which could be compelling ways to engage different communities (P-14; G-22; R-23; R-25). Such messaging should come from people ‘who are there, who are trusted, who are on the ground’ (R-24).

Consumers must be included as stakeholders in the development of any screening programme [C-10]. A dedicated consumer advisory panel with 10–12 consumers with diverse backgrounds could help “*surface the issues… [and create a] safe environment for them to be able to voice things*” [C-10]. Including 1–2 consumers on a board is unlikely to capture the diversity of consumer concerns and needs, and the medical professionals or academics may intimidate consumers to remain silent (C-10).

## Discussion

This is the first study to explore the perspectives of a wide range of Australian key stakeholders regarding screening for AF, and the idea of a national screening programme. We found broad support for AF screening as a concept, but many participants felt there is insufficient evidence to justify a systematic, national screening programme. There is concern that current opportunistic screening in Australia is neither effective nor capturing all cases of AF. Currently, the burden to screen lies with GPs, but this is not sustainable in the current landscape, especially as there is no remuneration. There was strong support for improving screening through enhancing opportunistic screening, which could be facilitated by alternate approaches either within the general practice setting, community or even self-screening. Regardless of the screening method, there were nine key recommendations identified which would need to be addressed in the design of an AF screening programme (box 1).

Box 1 **Policy and system level recommendations**

1. Address research gaps regarding the benefit of screening; cost effectiveness; and when to treat screen-detected atrial fibrillation (AF).
2. Identify relevant organisations and establish key responsibilities around governance and coordination of AF screening, as effective inter-agency collaboration is essential.
3. Identify how screening will be funded, and how remuneration can be delivered.
4. Work with key local organisations (including local Public Health Networks) to identify appropriate solutions which address local barriers such as workflow, with consideration given to solutions outside of general practice.
5. Establish how to reach the target population and ensure high-risk populations are included (such as Aboriginal communities and people with additional stroke risk factors).
6. Ensure that access to screening is equitable, especially for those who do not have a regular GP.
7. Drive consumer demand through developing consumer education which is culturally appropriate.
8. For solutions that use smartwatches or handheld devices, consider data security and the interoperability of data management systems and transfer to medical records.
9. Ensure that any screening solution includes a defined pathway for those detected with AF to access medical review and treatment.

GP, general practitioner.

The lack of stakeholder support for systematic screening is influenced by results from recent randomised control trials (including STROKESTOP^14^ and LOOP^15^ which were underpowered to show a reduction in stroke. Similarly, the UK National Screening Committee does not currently support screening because the evidence does not show a net benefit at a reasonable cost.^16^ These committee recommendations will be reassessed when large studies, including SAFER,^17^ provide definitive evidence regarding stroke reduction and cost benefit.^16^ If evidence supports systematic screening of all people ≥65 years, it is important to have already considered feasible solutions to implement. Centralisation of screening provides many benefits and addresses many of the key recommendations, especially clarity regarding the responsibilities for screening and ensuring everyone is offered screening. This contrasts with current opportunistic screening, which is dependent on available time and interest and hampered by access issues as many people cannot access regular medical care. Feasibility of centralised models has been demonstrated in the UK and Europe,^18^,^20^ with one study showing their model was cost-effective.^21^ Cost-effectiveness of population-based screening has also been demonstrated in Europe using Markov modelling.^22^ However, prior to consideration of a centralised model in Australia, an Australian-based economic analysis would be required.

Increasing current opportunistic screening was strongly supported by stakeholders. If opportunistic screening, rather than a centralised approach, is used, then a variety of different screening methods would likely be required across the country. Local health settings vary widely across Australia, so consideration needs to be given to available local resources and how solutions could integrate within the daily workflow in each setting. Numerous and varied opportunistic screening solutions have demonstrated feasibility for increasing the diagnosis of AF.^23^ Feasible solutions vary in detection method (eg, pulse palpation, handheld ECG or photoplethysmography, smart watches), location (eg, general practice, pharmacy, community settings) and who performs the screen (eg, GP, nurse, staff member, self-screening).^7 23^ Rather than investigating new methods to screen, we should consider which of the existing feasible methods suits the specific local context. The healthcare-level barriers of available time and staff workflow are evident in all screening solutions (regardless of who is performing the screen) and need to be considered when designing the solution.^24^,^26^ These key barriers consistently appear in the literature. The IDEAL-MD study^27^ found GPs screened only 10% of people in the intervention arm, rather than the expected 85%, due to time barriers and ‘loss of inertia’ to continue screening. Similar issues were experienced in the D2AF study.^28^ Careful planning to overcome barriers, defined roles and clear protocols at the practice level may assist this.^26 29^

Remuneration for time spent screening was one of the biggest barriers identified, consistent with previous studies.^27 28^ As previously stated, remuneration needs to be resolved at a systems level. Current remuneration options in Australia are limited to government, private health or user-pay models.^30^ If a private health or user-pay option is adopted, this would likely exacerbate health equity issues. Thus, government funding models should be explored. Within general practice, possible funding models include fee-for-service, capitation or pay-for-performance.^31^ Fee-for-service payments could also be extended to nursing and allied health. These models could work for either increasing opportunistic screening or a more systematised-targeted screening approach centred in general practice. Currently, there are only three national population-based screening programmes in Australia (ie, breast, bowel and cervical cancer).^32^ These successful programmes use a centralised approach and are funded from a combination of the Australian Government health budget and State/Territory budgets.^33^

Regardless of the screening approach, there is a need for more evidence around implementation strategies globally. ^34^ This is required to assist healthcare systems to overcome both system-level and local-level barriers. Currently, screening committees in the UK, USA and Canada do not include evidence-based implementation as a criterion to determine their recommendations. Viability of screening recommendations hinges on the ability and ease of implementation. Process evaluations of studies, with stakeholder interviews, provide this essential evidence. There is a need for process evaluations to be included in all large trials of AF screening. These evaluations will help guide implementation if the trials have a positive outcome.

The major strength of this work is that our study is one of only two studies which include perspectives of relevant stakeholders beyond consumers and health professionals.^35^ Given the range of issues that need to be addressed at a systems level, it is imperative to understand and incorporate the views of stakeholders who are involved in the governance of screening. It is important to note the limitations of our study which may impact the generalisability of our findings. Our study is based on the views of Australian stakeholders only. Consumers were not interviewed, so any inferences related to patients/consumers are from the perspective of non-consumer stakeholders. Findings are limited to the views of invited stakeholders who agreed to participate, which may introduce participation bias. Furthermore, it is likely that the views may not fully represent all of Australia which is diverse in culture and rurality. Australia has a complex multi-tiered system of healthcare governance, and we did not have representation from every region and level of government. However, it is important to note that our findings are consistent with those from other international qualitative research on AF screening,^27^,^2935 36^ and thus, our findings and recommendations are likely relevant to other countries and healthcare systems.

## Conclusions

Stakeholders broadly support the idea of AF screening as a concept. However, all stakeholders agree that further evidence is required prior to determining the most appropriate model for implementation of screening in Australia. Stakeholders envisaged a semisystematic approach tailored to specific health settings, rather than a formalised systematic national screening programme. All approaches will require a source of funding or remuneration, as it is thought that screening is unable to be incorporated into current workflows of health professionals. Evidence of feasibility is required for any solution. It is recommended that all future screening research include a process evaluation of implementation including qualitative interviews of the key stakeholders, as no solution will be successful unless it addresses the needs of all stakeholders.

## Supplementary material

10.1136/bmjopen-2025-109404 online supplemental file 1

10.1136/bmjopen-2025-109404 online supplemental file 2

## Data Availability

All data relevant to the study are included in the article or uploaded as supplementary information.

## References

[1] Kim J, Neville E, Dalli L (2024). on behalf of the Stroke Foundation, Economic Impact of Stroke 2024. Stroke Foundation.

[2] Kilkenny MF, Dalli LL, Kim J (2020). Factors Associated With 90-Day Readmission After Stroke or Transient Ischemic Attack: Linked Data From the Australian Stroke Clinical Registry. Stroke.

[3] Xian Y, Wu J, O’Brien EC (2015). Real world effectiveness of warfarin among ischemic stroke patients with atrial fibrillation: observational analysis from Patient-Centered Research into Outcomes Stroke Patients Prefer and Effectiveness Research (PROSPER) study. BMJ.

[4] Brieger D, Amerena J, NHFA CSANZ Atrial Fibrillation Guideline Working Group (2018). National Heart Foundation of Australia and the Cardiac Society of Australia and New Zealand: Australian Clinical Guidelines for the Diagnosis and Management of Atrial Fibrillation 2018. Heart Lung Circ.

[5] Hindricks G, Potpara T, Dagres N (2020). ESC Guidelines for the diagnosis and management of atrial fibrillation developed in collaboration with the European Association for Cardio-Thoracic Surgery (EACTS): The Task Force for the diagnosis and management of atrial fibrillation of the European Society of Cardiology (ESC) Developed with the special contribution of the European Heart Rhythm Association (EHRA) of the ESC. Eur Heart J.

[6] Chan N-Y, Orchard J, Agbayani M-J (2022). 2021 Asia Pacific Heart Rhythm Society (APHRS) practice guidance on atrial fibrillation screening. J Arrhythm.

[7] Wong KC, Nguyen TN, Chow CK (2024). Global implementation and evaluation of atrial fibrillation screening in the past two decades - a narrative review. *NPJ Cardiovasc Health*.

[8] Lowres N, Neubeck L, Salkeld G (2014). Feasibility and cost-effectiveness of stroke prevention through community screening for atrial fibrillation using iPhone ECG in pharmacies. The SEARCH-AF study. Thromb Haemost.

[9] Lyth J, Svennberg E, Bernfort L (2023). Cost-effectiveness of population screening for atrial fibrillation: the STROKESTOP study. Eur Heart J.

[10] Abolghasem Gorji H, Khosravi M, Mahmoodi R (2023). Cost-Effectiveness of Atrial Fibrillation Screening Strategies: A Systematic Review. Iran J Public Health.

[11] The Economist Intelligence Unit (2017). Preventing stroke: Uneven progress. A global policy research program.

[12] Braun V, Clarke V (2006). Using thematic analysis in psychology. Qual Res Psychol.

[13] Willig C (2013). Introducing qualitative research in psychology..

[14] Svennberg E, Friberg L, Frykman V (2021). Clinical outcomes in systematic screening for atrial fibrillation (STROKESTOP): a multicentre, parallel group, unmasked, randomised controlled trial. The Lancet.

[15] Svendsen JH, Diederichsen SZ, Højberg S (2021). Implantable loop recorder detection of atrial fibrillation to prevent stroke (The LOOP Study): a randomised controlled trial. The Lancet.

[16] UK National Screening Committee Adult screening program: atrial fibrillation 2019 recommendations.

[17] Mant J, Modi RN, Dymond A (2024). Randomised controlled trial of population screening for atrial fibrillation in people aged 70 years and over to reduce stroke: protocol for the SAFER trial. BMJ Open.

[18] Engdahl J, Andersson L, Mirskaya M (2013). Stepwise screening of atrial fibrillation in a 75-year-old population: implications for stroke prevention. Circulation.

[19] Mant J, Modi RN, Charlton P (2024). The feasibility of population screening for paroxysmal atrial fibrillation using hand-held electrocardiogram devices. *Europace*.

[20] Modi RN, Massou E, Charlton PH (2025). Screening for atrial fibrillation with or without general practice involvement: a controlled study. *BMC Prim Care*.

[21] Aronsson M, Svennberg E, Rosenqvist M (2015). Cost-effectiveness of mass screening for untreated atrial fibrillation using intermittent ECG recording. *Europace*.

[22] Bernfort L, Lyth J, Appelberg K (2026). Cost-effectiveness of atrial fibrillation screening programmes across European nations. Eur Heart J Qual Care Clin Outcomes.

[23] Wahab A, Nadarajah R, Larvin H (2025). Systematic screening for atrial fibrillation with non-invasive devices: a systematic review and meta-analysis. *Lancet Reg Health Eur*.

[24] McKenzie K, Lowres N, Orchard J (2022). Staff acceptability and patient usability of a self-screening kiosk for atrial fibrillation in general practice waiting rooms. *Cardiovasc Digit Health J*.

[25] Lowres N, Krass I, Neubeck L (2015). Atrial fibrillation screening in pharmacies using an iPhone ECG: a qualitative review of implementation. Int J Clin Pharm.

[26] Orchard J, Li J, Gallagher R (2019). Uptake of a primary care atrial fibrillation screening program (AF-SMART): a realist evaluation of implementation in metropolitan and rural general practice. BMC Fam Pract.

[27] Kaasenbrood F, Hollander M, de Bruijn SH (2020). Opportunistic screening versus usual care for diagnosing atrial fibrillation in general practice: a cluster randomised controlled trial. Br J Gen Pract.

[28] Uittenbogaart SB, Becker SJ, Hoogsteyns M (2022). Experiences with screening for atrial fibrillation: a qualitative study in general practice. BJGP Open.

[29] Theunissen LJHJ, Abdalrahim RBEM, Dekker LRC (2022). Regional implementation of atrial fibrillation screening: benefits and pitfalls. *Eur Heart J Digit Health*.

[30] Australian Institute of Health and Welfare 2024 report: Health system overview.

[31] Chen W, van Gool K, Wright M (2024). Understanding general practice funding models in Australia and beyond. Aust J Gen Pract.

[32] Australian Government Australian Institute of Health and Welfare.

[33] Shiell A, Garvey K, Kavanagh S (2024). How do we fund Public Health in Australia? How should we?. Aust N Z J Public Health.

[34] McKenzie K, Jacob A, Freedman B (2026). Key stakeholder views on atrial fibrillation screening: a systematic mixed-studies review and interpretive analysis. Europace.

[35] Engler D, Hanson CL, Desteghe L (2022). Feasible approaches and implementation challenges to atrial fibrillation screening: a qualitative study of stakeholder views in 11 European countries. BMJ Open.

[36] Vermunicht P, Grecu M, Deharo J-C (2023). General practitioners’ perceptions on opportunistic single-time point screening for atrial fibrillation: A European quantitative survey. Front Cardiovasc Med.

