# Supplementary material for: Australian key stakeholder views regarding implementation of atrial fibrillation screening: a qualitative evaluation
Source: BMJ Open. 2026 May 20;16(5):e109404. doi: 10.1136/bmjopen-2025-109404 (PMC13202094; doi:10.1136/bmjopen-2025-109404)
Supplement: online supplemental file 1 [file bmjopen-16-5-s001.pdf]

Key stakeholder interview topic guide

Introduction and Consent Process

- Researcher to introduce themselves and check understanding of trial and processes
- Written informed consent and permission to audio record the interview obtained from the participant

---

Your role in AF diagnosis and treatment

1. Can you tell me a little bit about your current role?

Prompt:

- *General responsibilities*
- *Experiences of or responsibilities for AF screening/diagnosis and treatment*
- *Other cardiovascular or relevant responsibilities*

Current approaches to AF detection and treatment

2. What are your views around current approaches to the detection and treatment of AF? (ensure cover both detection and treatment)

Prompts:

- *Local initiatives (e.g. practice/state level), commercial initiatives, professional organisations*
- *National policy, Australian guidelines etc*
- *What is working/not working?*
- *What could we be doing differently?*

A national screening programme for AF

3. In Australia, AF is currently detected through a variety of different approaches. What we don't have at the moment is a national screening programme for AF. What are your views on the need for a national screening programme?

Prompts:

- *Would you be in favour of it being introduced or not? For what reasons?*
- *Do you have any concerns/reservations about a national AF screening programme*? (e.g. screening harms, costs, diversion of resources)
- *What might the barriers and facilitators to delivering a national programme be? (both detection of AF and treatment for screen-detected cases). How might the barriers be addressed?*
- *Do you have any recommendations for a national roll out?*
- *Available evidence and potential need for more evidence*
- *Benefits of screening programme*
- *Potential harms of screening programme (significant? How much should they affect the decision to roll it out?)*
- *What do you think the views of patients/public are around AF screening?*
- *What is your sense of the attitudes of clinicians/clinical organisations to AF screening?*
- *Can you think of any group who would/ (not) be supportive of a national screening programme?*

Additional questions/prompts dependent on participant's organisation/area

<u>Screening researchers / professionals</u>
Prompts on the introduction of a national AF screening programme:

- *What concerns do you think you would have about it?*
- *What reception do you think it would have?*
- *Who would not support it?*
- *What advice would you give the SAFER-AUS team, if the trial is successful and national AF screening is shown to have benefit?*

<u>Charities: Atrial Fibrillation Alliance Australia / Australian National Heart Foundation</u>
How important is a national AF screening programme for [charity name]?
Prompts:

- *What do you think the greatest priority is for AF [treatment/detection]?*
  - *How important is screening within this?*
- *How does AF screening fit within your organisations mission?*

Your organisation has done much to campaign for greater awareness of AF and to increase detection rates. What reception has your work had with different groups?
Prompts:

- *With clinicians*
- *With politicians*
- *With policymakers*
- *With patients/the public*

<u>Professional clinical bodies + public organisations/Clinicians</u>
How important is AF screening for your organisation?
Prompts:

- *(Clinicians): What would it change/ what implications can you foresee for your work/your ambitions for improving patient care?*
- *How useful do you think it is to have more patients diagnosed with AF via screening?*
- *How important is AF screening for reducing stroke rates?*

<u>Clinicians</u>
The screening being trialled through SAFER-AUS involves participants using an ECG device four times a day for three weeks. It's therefore likely to pick up some cases of asymptomatic, paroxysmal AF. What do you think about this?
Prompts:

- *How beneficial is it to improve paroxysmal AF detection rates? Are these useful diagnoses? Are we 'scraping the barrel'?*
- *What impact do you think identifying this kind of patient would have on your work?*
- *To what extent do you think that identifying this kind of patient will improve stroke rates?*

Final Questions to cover

Can you think of anything else that we have not discussed, about this topic, which you would like to raise?

Is there anyone else it would be useful for us to speak to (an individual in your organisation/another organisation etc)?

Interview close

- Participants will be thanked for taking part in the trial and reminded that their responses will remain confidential
- Participants will once again have the opportunity to ask the researcher any questions and will also be given the opportunity to raise any additional issues that they feel are relevant to the topic
- Participants will be reminded that they can contact the research team at any time using the details on the information leaflet should they have any further questions or wish to remove their responses from the trial.
