# Supplementary material for: Australian key stakeholder views regarding implementation of atrial fibrillation screening: a qualitative evaluation
Source: BMJ Open. 2026 May 20;16(5):e109404. doi: 10.1136/bmjopen-2025-109404 (PMC13202094; doi:10.1136/bmjopen-2025-109404)
Supplement: online supplemental file 2 [file bmjopen-16-5-s002.docx]

**Reflexivity statement KM**

The research process involved interviews with key stakeholders about their perspectives on AF screening. Although I am an experienced qualitative researcher in public health, I am a relative outsider to the world of AF screening in Australia, and I do not have a background in health care such as general practice, nursing or pharmacy. My own beliefs about screening have to some extent been impacted by my personal experience. I do not have AF, or any other heart condition, and I do not know anyone who has had a stroke from undetected AF. However, I do have a significant medical condition that, without treatment, can lead to cancer. This condition was detected in the context of routine screening for another condition.

Going into the interviews, I reflected that my own non-expert beliefs about screening in general, and probably about AF, were that it was an obvious good, and the only question was how to get it to work in a way that was cost effective and not too onerous for health care staff or patients. I was able to take this belief into consideration during the interviews, and adopt a position of curiosity and questioning when participants held views about the value of screening that were not coherent with my own non-expert beliefs. I kept a notebook of my own responses to interviewees, in which I reflected on my own changing understanding of AF screening as the interviews progressed. This helped inform the data analysis, as I was able to reflect on and set aside any biases towards screening that I held at the start of the project.
