# Supplementary material for: Australian key stakeholder views regarding implementation of atrial fibrillation screening: a qualitative evaluation
Source: BMJ Open. 2026 May 20;16(5):e109404. doi: 10.1136/bmjopen-2025-109404 (PMC13202094; doi:10.1136/bmjopen-2025-109404)
Supplement: Supplementary file 3 [file bmjopen-2025-109404.reviewer_comments.pdf]

PEER REVIEW HISTORY

BMJ Open publishes all reviews undertaken for accepted manuscripts. Reviewers are asked to complete a checklist review form and are provided with free text boxes to elaborate on their assessment. These free text comments are reproduced below.

ARTICLE DETAILS

Title (Provisional)

Australian key stakeholder views regarding implementation of atrial fibrillation screening: a qualitative evaluation

Authors

Mckenzie, Kirsty; Freedman, Ben; Jacob, Anushka; Modi, Rakesh N; Lowres, Nicole

---

VERSION 1 - REVIEW

**Reviewer**	**1**
**Name**	**Lotto, Robyn**
**Affiliation**	**Liverpool John Moores University, School of Nursing and Allied Health**
**Date**	**11-Oct-2025**
**COI**	**None**

Thank you for inviting me to review this exciting paper. It reflects a well designed, inclusive study providing insight into an international problem.

I have some minor comments/questions.

Further detail around the sample in terms of diversity – ethnicity in particular – you provide a section around Aboriginal health but it is unclear whether they were represented within the study.

You discuss mapping but no real detail is provided about how this related to the sampling.

Further detail on “data saturation” would be useful – particularly in light of the arguments by Braun and Clarke (2022) against such a concept.

In terms of rigour, some form of reflexivity would be useful in particular around the analysis – inter code reliability, role of the researchers and potential biases.

Findings

Were there any conflicts/differences between groups? The findings don't currently reflect the input of multiple stakeholder groups.

Discussion

This is an international issue, and whilst Australia has its own peculiarities, more could be done to draw out international learning.

There is little around policy and system level implications – how might findings inform the design of a national program

Some redundancy between “Findings” and “Key recommendations” sections.

Again, this is well designed, well written and will be of interest to the readership.

Thank you

**Reviewer**	**2**
**Name**	**Jones, Nicholas**
**Affiliation**	**University of Oxford, Primary Care Health Sciences**
**Date**	**03-Feb-2026**
**COI**	**None**

Thanks for the opportunity to read this interesting paper, exploring the views of key Australian stakeholders regarding implementing AF screening. I appreciate the effort to explore the opinion of stakeholders that have not been well covered in previous research on this topic. It was also interesting to hear the perspective of screening among aboriginal communities as an important sub-group.

Major comments;

The participant groups mentioned in the abstract doesnt seem to align with the main text e.g. who represent patient support or public health? Very little information is given about the participants; it sounds as though a structured approach to identifying participants was taken with stakeholder mapping mentioned but more information would be extremely helpful. For example - two industry representatives are chosen but no information seems to be provided on who these were? Clearly - their interest in AF screening might be hugely impacted by this and they may have very different motivations to support screening or not. What are the different roles and experience of the healthcare professionals? Did you reach saturation of views through the interviews or was the sample pre-specified? Understanding more on the context of those interviewed and how selected would be crucial to understanding the results.

Linked to this - it seems interesting regarding who is making some of the comments and perhaps this could be highlighted in the text; as an example it seems the idea that GP prompts are needed (p7, line 24) is suggested by charities and professional bodies, not healthcare professionals who one might expect would suggest this if they felt it would help. The results are presented as though they were fairly homogenous across the different

stakeholder groups, perhaps the authors could comment on that if they feel that is true or else make clear if there were differences in feedback given between groups.

It's also interesting to reflect on the participants when considering their views on national screening. The only national screening approach recommended was for all people aged >=65 to attend their GP for screening (by single lead ECG?), yet this is not an approach tested in most of the AF screening RCTs nor one that is widely proposed. Does this reflect on the knowledge-base of the participants and if so, how well placed are they to consider the option of national screening? Did you consider outlining more feasible approaches to national screening to see if this could have prompted different views? I think you recognise this limitation in paragraph 3 of page 15 where you say centralised screening might be supported if the RCTs are positive, despite this not being recommended by your stakeholders.

The second paragraph on page 9 starts by that several participants could not see a downside of increased screening, yet this seems at odds with the next sentence where it reports most people mentioned at least one potential harm. It would be helpful to make this clear if possible.

The PPI section mentions this paper is about non-patient stakeholders, which is fine - I would be a bit cautious to make clear that the inferences about patients are therefore clearly described as the opinion of those interviewed e.g. page 12, line 55 "Consumers know little about AF" - this is quite a broad statement, albeit qualified in the second part of the paragraph.

The Discussion outlines the importance of renumeration for any screening work; the additional costs of screening has been recognised as a key reason not to screen by the National Screening Committee in the UK given this would mean funds being diverted from other areas that might be more cost-effective – I think this needs acknowledging in the discussion, perhaps by reflecting in how these stakeholders view align and disagree with screening committee recommendations on AF.

The only limitation given seems to be that the study is based on views of Australian stakeholders only. Do the authors think there might be other limitations that would be worth discussing such as potential bias among the participants, issues of data saturation, incomplete knowledge of options for AF screening. Are the views representative of all Australia? Might federal v national differences be relevant that are not discussed? Screening might be delivered in many forms and among many populations and this context is important when considering some of the issues raised e.g. cost-effectiveness. The broad discussion around screening might be considered a limitation as the granular detail by focused discussion on practical screening approaches could have provided more specific feedback from the stakeholders.

Minor

Please can you provide the identifier of the participant for each quote - even if small eg. 'a little bit of AF'.

As a non-Australian, I feel some of the context described is not entirely clear. For example, how important is it that there is no renumeration for AF screening under medicare in terms of the proportion of the population this applies to? Presumably a new case of AF detected by screening would be renumerated?

The author group has an outstanding track record in this field and have referenced some of their excellent work in this field - though valuable it might be helpful to include other relevant research in the field e.g. recent Lancet systematic review of screening approaches (10.1016/j.lanepe.2025.101298) or a recent paper on cost-effectiveness of AF screening (DOI: 10.1093/ehjqcco/qcaf099). Discussing limitations of a single-lead ECG approach could be made with reference to VITAL-AF or IDEAL-MD

VERSION 1 - AUTHOR RESPONSE

Comments	Response
**Reviewer 1:**	
Thank you for inviting me to review this exciting paper. It reflects a well-designed, inclusive study providing insight into an international problem. I have some minor comments/questions.<br>Again, this is well designed, well written and will be of interest to the readership.<br>Thank you	
1. Further detail around the sample in terms of diversity – ethnicity in particular – you provide a section around Aboriginal health but it is unclear whether they were represented within the study.	**Please refer to response to Editor comment 4 above.**
2. You discuss mapping but no real detail is provided about how this related to the sampling.	**Please refer to response to Editor comment 3 above.**
3. Further detail on “data saturation” would be useful – particularly in light of the arguments by Braun and Clarke (2022) against such a concept.	**Thank you for highlighting this error. We note that ‘data saturation’ was mentioned by error. This error was only mentioned in the Strengths and limitations key points, and did not occur anywhere else in the manuscript. This wording has now been removed.**<br><br>**In response to this comment, and Editor comment 1, we have revised the “strengths and limitations section” (Please refer to response to Editor comment 1)**

	**In addition, we have also revised the Methods, Participants section to provide more details on the recruitment process (Please refer to response to Editor Comment 3).**<br>*"We continued to invite stakeholders until we had adequate representation across the six broad stakeholder groups. In total 45 stakeholders were invited and 25 agreed to participate."*
4. In terms of rigour, some form of reflexivity would be useful in particular around the analysis – inter code reliability, role of the researchers and potential biases.	**We have addressed this issue by revising the Methods, Analysis section as follows:**<br><br>*Analysis*<br>*The interviews were transcribed verbatim and analysed using an iterative thematic analysis approach12. Coding was undertaken by KM. Coding was driven by a pre-existing framework derived from the research questions and an inductive approach in which novel groupings of information were identified from the data[13]. Each transcript was coded line-by-line based on identifying similar concepts, ideas and patterns in the data, and concerning the research questions. Attention was given to whether deviant cases (uncommonly expressed ideas) might be important. Once all transcripts were coded, the research team met to discuss the working coding structure and group these into themes. To ensure that results reflected participants' views and were not overly simplified, emerging themes were checked against the transcripts. Various measures were taken to reduce bias. During the initial coding phase, a subset of transcripts was also read by NL, and KM and NL met to compare their response to the data and refine the coding structure. KM also kept notes on her responses to participants perspectives, which in cases differed from her own so these could be taken into account during coding (see Supplement 2: Reflexivity statement). After themes were finalised, they were developed into a narrative, which both explained the themes and provided evidence from the data in the form of quotations.*<br><br>**A reflexivity statement has also been provided as Supplementary Data.**
5. Findings<br>Were there any conflicts/differences between groups? The findings don't currently reflect the input of multiple stakeholder groups.	**We appreciate the comment on the homogeneity of the findings between groups. In analysing the data, we were attentive to differences in perspectives between stakeholder groups. However, for most findings, there was broad agreement between groups on the existing challenges, needs, and potential solutions. We also note that our**

	**samples for some groups were quite small (industry and government), and that roughly a third of our sample was in more than one stakeholder group – these factors make granular differentiation by stakeholder group methodologically difficult.**<br><br>**We have therefore revised the results section extensively, to reflect stakeholder homogeneity, and where appropriate, areas where groups held views more strongly. Each result is followed by the ID of all the stakeholders who expressed that idea. We have included overarching statements throughout to explicitly state which stakeholders agreed with the concept and which did not.**<br><br>*Example:*<br>*However, participants from all stakeholder groups expressed concerns about undetected AF in the general population, and there was agreement across all stakeholder groups that many GPs were not opportunistically screening [I-01; C-02; H-05; C-06; C-07; P-08; G/R-13; P-14; H/P-19; H-21; G-22; R-25].*<br><br>**We have prefaced the results section, second paragraph with the following statement:**<br><br>*In analysing the data, we were attentive to differences in perspective between stakeholder groups. However, for most findings, there was broad agreement between groups on the existing challenges, needs, and potential solutions. A decision was therefore made to not differentiate findings systematically by stakeholder group but to make reference where appropriate to views that were expressed more strongly by specific stakeholder groups.*
6. Discussion<br>This is an international issue, and whilst Australia has its own peculiarities, more could be done to draw out international learning.	**In response to the comments from Reviewer 1 and 2, we have significantly restructured and revised the entire discussion section.**<br><br>**To draw out international learning:**<br>• We have added a lot more comparison of our results to international studies and literature in paragraphs 2-6, which demonstrates the universal nature of the results<br>• We framed the results in relation to International screening committee guidelines and evidence in paragraph 2 and 5

	• We have stated how the results are relevant beyond the Australian setting in paragraph 6
7. There is little around policy and system level implications – how might findings inform the design of a national program	**To draw out policy and system level implications:**<br>• We have summarised the 9 policy and system level implications in Box 1 in the discussion.<br>• We have revised the text so that it is clear we are talking about system level implications in paragraph 2, 4 and 5
8. Some redundancy between "Findings" and "Key recommendations" sections.	**We appreciate the opportunity to address this important point in the manuscript.**<br><br>**To reduce redundancy:**<br>• We have removed the key recommendations from the findings/results section.<br>• We have completely revised the discussion<br>• We have summarized the 9 key policy and system recommendations in the discussion
**Reviewer 2:**	
Thanks for the opportunity to read this interesting paper, exploring the views of key Australian stakeholders regarding implementing AF screening. I appreciate the effort to explore the opinion of stakeholders that have not been well covered in previous research on this topic. It was also interesting to hear the perspective of screening among aboriginal communities as an important sub-group.	
1. The participant groups mentioned in the abstract doesnt seem to align with the main text e.g. who represent patient support or public health?	**We thank the reviewer for identifying this error. We originally started with 10 broad stakeholder groups (as outlined in the abstract) and then realised the high potential for re-identification of participants with this level of stratification when writing the manuscript. We therefore re-classified our groupings to six groups for the purpose of the manuscript and de-identification of the participants. The abstract has now been revised to reflect the correct groupings and the stakeholder mapping has now been provided (as per the response to Editor comment 3).**
2. Very little information is given about the participants; it sounds as though a structured approach to identifying participants was taken with stakeholder mapping mentioned but more information would be extremely helpful. For example - two industry representatives are chosen but	**Please refer to response to Editor comment 3 and 4 above.**<br><br>**In relation to saturation of views, please refer to the response to Reviewer 1, comment 3. Sampling was not based on data saturation. We ceased sending invitations to participants once we had adequate representation across**

no information seems to be provided on who these were? Clearly - their interest in AF screening might be hugely impacted by this and they may have very different motivations to support screening or not. What are the different roles and experience of the healthcare professionals? Did you reach saturation of views through the interviews or was the sample pre-specified? Understanding more on the context of those interviewed and how selected would be crucial to understanding the results.	**the stakeholder groups. 25 stakeholders agreed from the 45 invitations sent.**
3. Linked to this - it seems interesting regarding who is making some of the comments and perhaps this could be highlighted in the text; as an example it seems the idea that GP prompts are needed (p7, line 24) is suggested by charities and professional bodies, not healthcare professionals who one might expect would suggest this if they felt it would help.	**In response to this comment and Reviewer 1 comment 5, we have made substantial revisions to the manuscript to identify where there is consensus between stakeholder groups, and where some groups express concerns more than others. (Please refer to our response to Reviewer 1, comment 5)**<br><br>**This has included extensive revision of the results section "The current context, Opportunistic screening is ad hoc and fragmented and dependent on GP capacity" which amalgamates and improves the clarity about the issues for screening in general practice.**
4. The results are presented as though they were fairly homogenous across the different stakeholder groups, perhaps the authors could comment on that if they feel that is true or else make clear if there were differences in feedback given between groups.	**Please refer to response to Reviewer 1 comment 5 above.**
5. It's also interesting to reflect on the participants when considering their views on national screening. The only national screening approach recommended was for all people aged >=65 to attend their GP for screening (by single lead ECG?), yet this is not an approach tested in most of the AF screening RCTs nor one that is widely proposed. Does this reflect on the knowledge-base of the	**We appreciate the opportunity to improve the clarity of our results section in relation to this issue. We have significantly revised the results around systematic screening, evidence and what participants recommended to improve the clarity, especially the "**_The need for evidence to justify a national systematic screening program"_ **section, which has completely been rewritten.**<br><br>**We have also significantly revised the discussion to address this, in particular Paragraph 2.**

participants and if so, how well placed are they to consider the option of national screening? Did you consider outlining more feasible approaches to national screening to see if this could have prompted different views? I think you recognise this limitation in paragraph 3 of page 15 where you say centralised screening might be supported if the RCTs are positive, despite this not being recommended by your stakeholders.	• Paragraph 2 more clearly discusses the international evidence and how this may impact stakeholder views<br>• Paragraph 2 also discusses the views of stakeholders in relation to the UK screening committee recommendations, and how this may be altered once the UK screening committee reviews it's recommendations if forthcoming RCTs are positive.
6. The second paragraph on page 9 starts by that several participants could not see a downside of increased screening, yet this seems at odds with the next sentence where it reports most people mentioned at least one potential harm. It would be helpful to make this clear if possible.	**We recognise that the statement about "several participants could not see a downside of increased screening" is not clear. As part of the extensive revision of the results sections, this sentence has been removed to improve clarity.**
7. The PPI section mentions this paper is about non-patient stakeholders, which is fine - I would be a bit cautious to make clear that the inferences about patients are therefore clearly described as the opinion of those interviewed e.g. page 12, line 55 "Consumers know little about AF" - this is quite a broad statement, albeit qualified in the second part of the paragraph.	**We have revised the manuscript throughout to make it clear that inferences about patients are the views of non-consumer stakeholders. Example edit:**<br><br>*Some participants commented that consumers know little about AF, and health literacy in general is very low in some communities [C-10; H/P-19; R-23; R-24].*<br><br>**We have also mentioned this specifically in the limitations section:**<br><br>*Consumers were not interviewed, so any inferences related to patients/consumers are from the perspective of non-consumer stakeholders.*
8. The Discussion outlines the importance of renumeration for any screening work; the additional costs of screening has been recognised as a key reason not to screen by the National Screening Committee in the UK given this would mean funds being diverted from other areas that might be more cost-effective – I think this needs acknowledging in the discussion, perhaps by reflecting in how these stakeholders view align	**To address this important point, we have revised the discussion to specifically include discussion on how the stakeholder views align with UK national screening committee recommendations in paragraph 2, as follows:**<br><br>*The lack of stakeholder support for systematic screening is likely influenced by results from recent randomised control trials (including STROKESTOP[14] and LOOP[15]) which were underpowered to show a reduction in stroke. Similarly, the UK National Screening Committee does not currently support screening because the evidence does not show a net benefit at a*

	and disagree with screening committee recommendations on AF.	*reasonable cost[16]. These committee recommendations will be reassessed when large studies, including SAFER[17], provide definitive evidence regarding stroke reduction and cost benefit[16]. If evidence supports systematic screening of all people ≥65 years, it is important to have already considered feasible solutions to implement. Centralisation of screening provides many benefits and addresses many of the key recommendations, especially clarity regarding the responsibilities for screening, and ensuring everyone is offered screening. This contrasts with current opportunistic screening, which is dependent on available time and interest, and hampered by access issues as many people cannot access regular medical care. Feasibility of centralised models has been demonstrated in the United Kingdom and Europe[18-20], with one study showing their model was cost-effective[21]. Cost-effectiveness of population-based screening has also been demonstrated in Europe using Markov modelling[22]. However, prior to consideration of a centralised model in Australia, an Australian-based economic analyses would be required.*
9.	The only limitation given seems to be that the study is based on views of Australian stakeholders only. Do the authors think there might be other limitations that would be worth discussing such as potential bias among the participants, issues of data saturation, incomplete knowledge of options for AF screening. Are the views representative of all Australia? Might federal v national differences be relevant that are not discussed? Screening might be delivered in many forms and among many populations and this context is important when considering some of the issues raised e.g. cost-effectiveness. The broad discussion around screening might be considered a limitation as the granular detail by focused discussion on practical screening approaches could have provided more specific feedback from the stakeholders.	**In response to this comment, we have made revisions to the "Discussion, Limitations section" which now includes the points raised by the reviewer, as follows:**<br><br>*It is important to note the limitations of our study. Our study is based on the views of Australian stakeholders only. Consumers were not interviewed, so any inferences related to patients/consumers are from the perspective of non-consumer stakeholders. Findings are limited to the views of invited stakeholders who agreed to participate, which may introduce participation bias. Furthermore, it is likely that the views may not fully represent all of Australia which is diverse in culture, and rurality. Australia has a complex multi-tiered system of health-care governance, and we did not have representation from every region and level of government. These factors limit the generalisability of our findings, however, our findings align with those from other international and local qualitative research on AF screening[20,30,31].*

10. Please can you provide the identifier of the participant for each quote - even if small eg. 'a little bit of AF'.	**We have gone through the manuscript and ensured that all quotes, even if they are small, have a participant identifier.**
11. As a non-Australian, I feel some of the context described is not entirely clear. For example, how important is it that there is no renumeration for AF screening under medicare in terms of the proportion of the population this applies to? Presumably a new case of AF detected by screening would be renumerated?	**To address this point, we have added additional context to the results, General practice-based screening section, paragraph 4:**<br><br>*Remuneration was mentioned by most stakeholders. There is no current remuneration for AF screening under the Medicare Benefits Schedule, and some mentioned the need for a new Medicare item number. Medicare item numbers identify specific medical services or diagnostic procedures that are eligible for a government subsidy. This would mean the cost of screening would be covered by the government rather than the patient.*
12. The author group has an outstanding track record in this field and have referenced some of their excellent work in this field - though valuable it might be helpful to include other relevant research in the field e.g. recent Lancet systematic review of screening approaches (10.1016/j.lanepe.2025.101298) or a recent paper on cost-effectiveness of AF screening (DOI: 10.1093/ehjqcco/qcaf099). Discussing limitations of a single-lead ECG approach could be made with reference to VITAL-AF or IDEAL-MD	**We have taken the opportunity to improve the manuscript with additional comparison and reflection on important international studies.**<br><br>**Throughout our revised discussion we have discussed the suggested additional papers and other relevant international studies, including:** Bernfort 2026, Wahab 2025, Vermunict 2023, Kaasenbrood 2020, Theunissen 2022, and Uittenbogaart 2023

VERSION 2 - REVIEW

Reviewer **1**

Name **Lotto, Robyn**

Affiliation **Liverpool John Moores University, School of Nursing and Allied Health**

Date **20-Mar-2026**

COI

Thank you. I really enjoyed reviewing your paper. You have attended to all comments thoroughly.

I have one minor comment - line 51 - although you have rephrased slightly, it could be more succinct. Could you replace pg 12 line 51 " Some participants commented that consumers know little about AF," with "some participants perceived that consumer awareness of AF is generally low, with some suggesting that health literacy is limited in certain communities" I feel this removes the over- generalisation risk.

Thank you

VERSION 2 - AUTHOR RESPONSE

Reviewer 1 comment:
Thank you. I really enjoyed reviewing your paper. You have attended to all comments thoroughly.
I have one minor comment - line 51 - although you have rephrased slightly, it could be more succinct. Could you replace pg 12 line 51 " Some participants commented that consumers know little about AF," with "some participants perceived that consumer awareness of AF is generally low, with some suggesting that health literacy is limited in certain communities" I feel this removes the over- generalisation risk.

Response:
We have amended the manuscript with the revised text, verbatim as suggested by Reviewer 1.
